# Oncological evaluation in the perioperative period using cfDNA with *BRAF* V600E mutation in patients with colorectal cancer

**DOI:** 10.1038/s41598-021-92795-8

**Published:** 2021-06-24

**Authors:** Keita Tanaka, Yoichiro Yoshida, Teppei Yamada, Takaomi Hayashi, Hideki Shimaoka, Fumihiro Yoshimura, Ryuji Kajitani, Taro Munechika, Yoshiko Matsumoto, Hideki Nagano, Akira Komono, Ryohei Sakamoto, Ryo Nakashima, Naoya Aisu, Gumpei Yoshimatsu, Suguru Hasegawa

**Affiliations:** Department of Gastroenterological Surgery, Fukuoka University Faculty of Medicine, 7-45-1 Nanakuma, Jonan-ku, Fukuoka, 814-0180 Japan

**Keywords:** Colorectal cancer, Surgical oncology

## Abstract

The detection of circulating cell-free DNA (cfDNA) by liquid biopsy is reported to provide prognostic information in colorectal cancer (CRC). Although the frequency of *BRAF* V600E mutation in CRC is less than 10%, it is associated with poor responses to conventional chemotherapy. We conducted a prospective study to investigate the relationship between the perioperative mutant allele frequency (MAF) of *BRAF* V600E and tumor recurrence, and to evaluate the possibility of early detection of recurrence. Among 362 patients who underwent radical resection, cfDNA was extracted from the perioperative blood of 11 CRC patients with *BRAF* V600E mutation and analyzed using the digital polymerase chain reaction (dPCR) system. The median follow-up time was 22 months, and there were four cases of recurrence. Although there was no correlation between recurrence and the perioperative MAF of *BRAF* V600E, tumor diameter was correlated with the MAF (p = 0.024), and the MAF increased with time in two patients from whom additional samples were obtained prior to recurrence. In this study, we identified a correlation between the pathological tumor diameter and the MAF, but it was difficult to predict recurrence by measuring cfDNA with *BRAF* V600E mutation in the perioperative period of radical resection of CRC.

## Introduction

Colorectal cancer (CRC) is the third most common cancer and the fourth most frequent cause of cancer-related death worldwide^1^. The standard treatment of CRC without metastasis is radical resection, adjuvant chemotherapy depending on cancer stage, and follow-up according to current guidelines. Despite recent advances in the management of CRC, it is estimated that approximately 30% of patients develop metastases and finally die of metastatic disease after radical resection^2–4^. Therefore, the early detection of patients at risk of developing metastatic disease after radical resection is critical to improving clinical outcome.

*BRAF* encodes a cytoplasmic serine/threonine kinase, and acts directly downstream of KRAS in the MAPK signaling pathway. *BRAF* mutations occur in approximately 3%–10% of CRC^5–8^. More than 90% of activating *BRAF* mutations in CRC are caused by a change in nucleotide 1799 of exon 15, which induces a thymine to adenine change, leading to a substitution of valine by glutamate. This mutation is recognized as tumors with *BRAF* V600E^9,10^. CRC patients harboring tumors with *BRAF* V600E have poor outcomes and a low response to conventional chemotherapy^11^. The recent approval of *BRAF* inhibitors for CRC has improved cancer treatment outcomes. If liquid biopsy can be used to select the need for postoperative adjuvant chemotherapy, the outcomes of *BRAF*-mutant CRC will be further improved. Although *BRAF* inhibitors have recently entered clinical use for treating *BRAF*-mutant cancers, cancers are known to have spatial and temporal heterogeneity, and mutations not identified in primary tumors have been shown to exist in metastatic tumors^12,13^. Therefore, it is desirable to monitor various genetic information in real time.

The term liquid biopsy was originally introduced as to the analysis of circulating tumor cells (CTCs)^14^. At present, liquid biopsy refers to a collective term that refers to the analysis of cancer-derived biomarkers isolated from the fluids of cancer patients^15^.There are a pool of cells and/or cell products derived from a primary or metastatic tumor, including CTCs, circulating cell-free DNA (cfDNA) or RNA, and exosomes in the peripheral blood of a cancer patient^16^. Analysis of these liquid biopsies can provide comprehensive real-time information of the tumor-associated changes in an individual cancer patient. These data can be used for the early detection of cancer, prognostic information (the risk for metastatic recurrence or progression), predictive information (sensitivity to anti-cancer agents), monitoring of treatment response, and identification of minimal residual disease (MRD)^17^. In terms of CTCs, they are more sensitive than tumor markers, and they can be used to predict prognosis when combined with tumor markers^18^. The presence of postoperative CTCs predicts decreased disease-free survival^19^, and the presence of preoperative CTCs predict early recurrence and decreased disease-free survival^20^. A review by Peach et al. summarized that the presence of CTCs in peripheral blood at least 24 h after tumor resection was an independent prognostic marker of recurrence^21^. However, there are no reports of the correlation between perioperative changes in cfDNA with *BRAF* V600E and prognosis.

In this study, we evaluated the dynamics of *BRAF* V600E mutation during the perioperative period by liquid biopsy to test the hypothesis that measurement of *BRAF* V600E in cfDNA in the early postoperative period is useful for predicting recurrence.

## Results

### Patient characteristics

**Figure 1:**
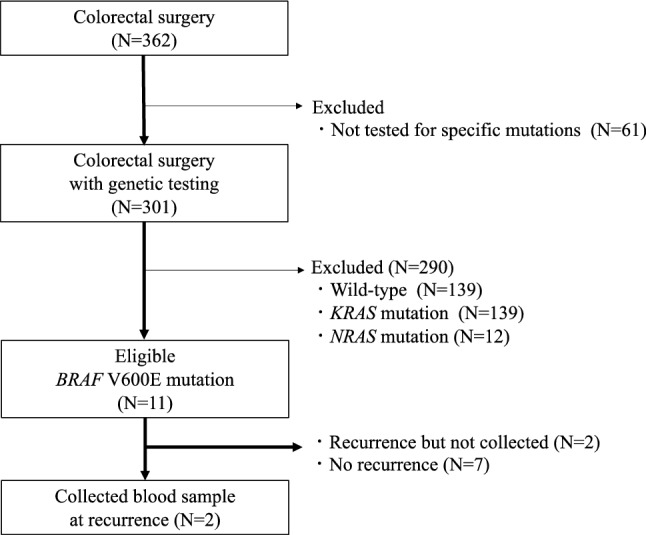
Flow chart presenting *BRAF* V600E mutation detection in the study. *N* number of patients.

A total of 362 patients underwent surgery for CRC at our hospital between April 2018 and March 2020. Sixty-one patients who did not undergo genetic testing for *KRAS*/*NRAS*/*BRAF* were excluded, and 301 patients were included in the study. There were 139 patients with *KRAS* mutations and 12 with *NRAS* mutations, and 139 were wild-type. Among the 151 patients with *KRAS* or *NRAS* mutations, none carried the *BRAF* V600E mutation. *BRAF* V600E mutation was identified in 11 (3.7%) cases using resected specimens. All patients had complete blood samples and underwent molecular analysis. We were able to collect samples from two of the four patients with recurrence after 30 days postoperatively, and molecular analysis was performed (Fig. 1).

The characteristics of these 11 cases are shown in Table 1. The median age was 69 years (range, 46–83), and seven cases were in women. The median body mass index was 21.6 kg/m^2^. The Eastern Cooperative Oncology Group performance score (ECOG–PS) was 0 in six patients, 1 in two patients, and ≥ 2 in three patients. In one case, duodenal NET was recognized in synchrony, and simultaneous resection was performed by laparotomy. All other cases underwent laparoscopic surgery. The median diameter of the pathological primary tumor at the baseline was 45 mm (range: 30–80 mm). Pathologically, four patients had lymph node metastasis, and seven patients did not.

**Table 1:** Demographics and characteristics of patients with *BRAF* V600E mutation.

Characteristic	Classification	N = 11
Sex	Male	4
	Female	7
Age (years)	Median (range)	69 (46–83)
BMI (kg/m^2^)	< 20	2
	20–24	7
	> 24	2
ECOG performance status	0	6
	1	2
	≥ 2	3
Primary tumor location	Right-sided	8
	Left-sided	3
Histological grade	Well	3
	Moderately	5
	Poorly	1
	Mucinous	2
p-Tumor depth	T1	0
	T2	2
	T3	5
	T4 (T4a/T4b)	4 (4/0)
p-Lymph node metastasis	N0	4
	N1 (N1a/N1b/N1c)	5 (3/2/0)
	N2 (N2a/N2b)	2 (0/2)
Recurrence site	Liver	1
	Peritoneum	3
Tumor marker	Pre-CEA (ng/ml)	4.6 (1.5–28.8)
	Pre-CA19-9 (U/ml)	7 (2–199)

*BMI* body mass index, *ECOG* Eastern Cooperative Oncology Group.

The progress of each patient is shown in Table 2. The mean observation period was 22 months. Five patients received postoperative adjuvant chemotherapy. Recurrence was observed in four cases. The recurrent organs were liver in one case and peritoneum in three cases. A patient with liver metastases died 17.2 months after treatment with liver resection and subsequent chemotherapy. Of the three patients with peritoneal disseminated metastases, one died at 9.4 months after surgery due to poor performance score (PS) and the inability to undergo chemotherapy. The remaining two patients were treated with chemotherapy, one of whom was resected for peritoneal dissemination and is still undergoing chemotherapy, and one of whom died at 11.6 months after surgery due to tumor progression.

**Table 2:** Progress of the patients’ treatment.

	Sex	Age	Location	Histological grade	Pre-CEA	Pre-CA19-9	Tumor size (mm)	P-tumor depth	P-lymph node metastasis	pStage	Pre-MAF (%)	Adjuvant therapy	Recurrence	DFS (mo)	Additional treatment	OS (mo)	Outcome
1	F	82	Cecum	Moderately	1.7	2	30	T2	N0	I	0.178	−	−	22.5	−	22.5	Alive
2	F	76	Ascending	Mucinous	14	7	80	T3	N0	IIA	1.622	−	−	22.8	−	22.8	Alive
3	F	70	Transverse	Moderately	4.6	7	50	T3	N0	IIA	0.266	−	−	22.4	−	22.4	Alive
4	F	77	Descending	Well	4.6	3	35	T4a	N0	IIB	0.673	−	P	7.2	−	9.4	Death
5	M	52	Ascending	Mucinous	6.3	6	30	T2	N1a	IIA	1.444	+	−	15.4	−	15.4	Alive
6	F	57	Ascending	Moderately	20.3	45	70	T3	N1a	IIB	3.659	+	−	22.4	−	22.4	Alive
7	F	83	Rectum	Moderately	1.5	12	45	T3	N1b	IIB	3.534	−	−	20.0	−	20.0	Alive
8	M	46	Transverse	Well	2	6	45	T3	N1a	IIB	0	+	−	18.6	−	18.6	Alive
9	M	52	Ascending	Moderately	2.2	33	65	T4a	N1b	IIB	4.626	+	P	5.2	C	11.6	Death
10	F	69	Ascending	Well	28.8	199	40	T4a	N2b	IIC	0.958	+	P	3.9	C, S	23.6	Alive
11	M	58	Ascending	Moderately	1.8	5	40	T4a	N2b	IIC	0.0998	−	Liver	2.7	C, S	17.2	Death

*DFS* disease-free survival, *OS* overall survival, *P* peritoneal dissemination, *C* chemotherapy, *S* surgery.

### Relationship between mutant allele frequency of *BRAF* V600E in the perioperative period and recurrence

**Figure 2:**
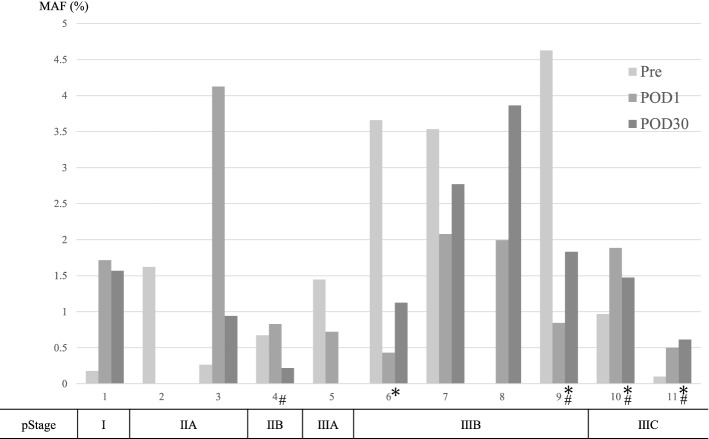
The mutant allele frequencies of *BRAF* V600E mutation in all cases. Perioperative changes in MAF were not related to the time course. The amounts of POD1 and POD7 in Case 2, POD30 in Case 5, and preoperative *BRAF* V600E in Case 8 were small and undetectable. The pStage is presented as described in the TNM 8 version. ^#^Recurrence; *Adjuvant chemotherapy; Pre, preoperative; POD, postoperative day.

We examined *BRAF* V600E in the perioperative cfDNA of all patients. The MAFs of *BRAF* V600E in all cases are shown in Fig. 2. Some patients had a high MAF even if they had not relapsed, while others had a low MAF but had relapsed. Changes in MAF did not fluctuate over time. The relationship between detection of *BRAF* V600E and recurrence was simplified using a cutoff value of 0.15% for MAF^22^ (Table 3). In patients who experienced relapse, the *BRAF* V600E mutation was detected in all postoperative measurements. Conversely, the mutation was inconsistently detected in patients who did not experience relapse. There was no correlation between cfDNA in the perioperative period and recurrence.

**Table 3:** Relationship between *BRAF* V600E detection and recurrence.

	Preoperative	POD1	POD30
Recurrence (−) N = 7	6/7 (85.7%)	6/7 (85.7%)	5/7 (71.4%)
Recurrence (+) N = 4	3/4 (75%)	4/4 (100%)	4/4 (100%)

The cutoff value of the mutant allele frequency was 0.15%.

*POD* postoperative day.

**Figure 3:**
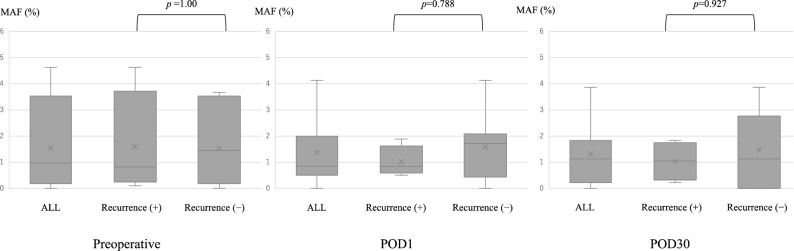
Relationship between the perioperative mutant allele frequency of *BRAF* V600E and recurrence. There was no significant difference between MAF preoperatively and on postoperative day (POD)1 and POD30 between recurrent and non-recurrent cases (Mann–Whitney U test; *p* = 1.00, *p* = 0.788, and *p* = 0.927, respectively).

**Figure 4:**
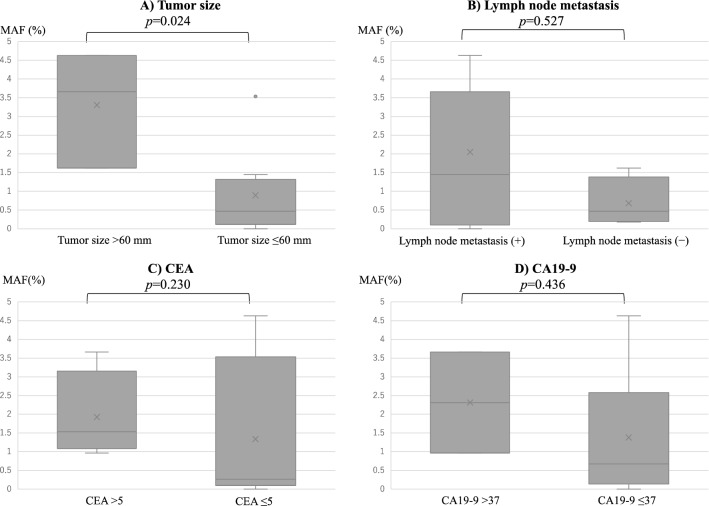
Relationship between preoperative *BRAF* V600E mutant allele frequency and tumor size, lymph node metastasis, and tumor markers. (**A**) Tumor size, (**B**) lymph node metastasis, (**C**) CEA, and (**D**) CA19-9. Pairwise comparisons showed that the larger the tumor size, the higher the MAF **(**Mann–Whitney U test; *p* = 0.024). There were no significant differences in the presence or absence of lymph node metastasis, CEA level, or CA19-9 level. (Mann–Whitney U test; *p* = 0.527, *p* = 0.230, and *p* = 0.436, respectively.)

A comparison of the perioperative MAF of *BRAF* V600E with recurrence is shown in Fig. 3. There was no significant difference in recurrence and MAF preoperatively or on postoperative days 1 or 30. The preoperative *BRAF* V600E MAF and each factor were compared (Fig. 4). Tumor diameter, lymph node metastasis, preoperative CEA, and preoperative CA19-9 were examined; the MAF was significantly higher in patients with tumor diameters of 60 mm or more.

### MAF of *BRAF* V600E at diagnosis of recurrence

**Figure 5:**
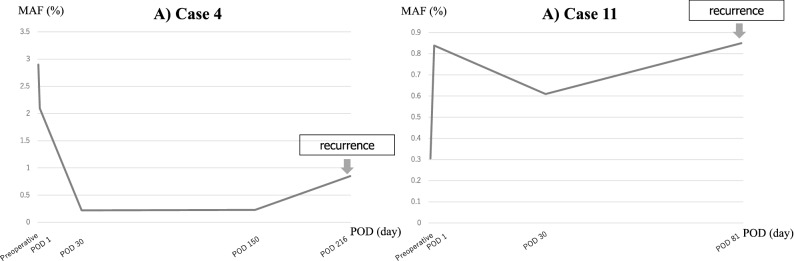
The mutant allele frequency of the *BRAF* V600E mutation was assessed in Case 4 and 11 until recurrence. In both cases, the cfDNA levels tended to increase over time. *POD* postoperative day.

We were able to collect a liquid biopsy from two cases of recurrence (case 4 and case 11) until the recurrence was confirmed on imaging. Case 4 had poor PS and could not receive postoperative adjuvant chemotherapy. At 7.2 months postoperatively, peritoneal dissemination was diagnosed. The MAF was 0.22% at 30 days postoperatively, 0.23% at 5 months postoperatively, and 0.85% at diagnosis of recurrence. However, case 11 had idiopathic thrombocytopenic purpura and could not receive adjuvant chemotherapy. At 2.7 months after surgery, liver metastasis was diagnosed. The MAF on postoperative day 30 was 0.61%, while the MAF at the start of chemotherapy was 0.85%. In both cases, the cfDNA levels showed an increasing trend over time (Fig. 5).

## Discussion

Recently, Zmrzljak et al. reported on the detection of the *BRAF* V600E mutation by liquid biopsy in patients with CRC before surgery^23^. To the best of our knowledge, this is the first prospective study to evaluate the oncological changes of perioperative cfDNA levels patients with the *BRAF* V600E mutation over time. In the present study, we showed two main findings. First, it was difficult to predict recurrence by measuring cfDNA with *BRAF* V600E in the perioperative period. Second, the perioperative MAF of *BRAF* V600E was not correlated with tumor markers or lymph node metastasis but was correlated with tumor diameter.

Previous reports demonstrated that cfDNA is a prognostic marker in metastatic CRC patients^24–26^. However, these studies were conducted in patients already diagnosed with distant metastasis. There have been reports that preoperative and pre-chemotherapy cfDNA levels are useful prognostic markers, but no mutation-specific reports have been published^27–29^. Although Shimada et al. reported that *BRAF* V600E positivity in resection specimens is an important molecular marker for predicting prognosis and the feasibility conversion surgery (conversion surgery defined as a surgical treatment aiming at a curable intention after tumors initially deemed technically or oncologically unresectable or only marginally resectable respond to chemotherapy) in patients with stage IV CRC^30^, no report describe the early prediction of recurrence using cfDNA containing *BRAF* V600E in the perioperative period after radical surgery. In this study, we examined the MAF of *BRAF* V600E preoperatively and on postoperative days 1 and 30 and found no correlation between MAF and recurrence. Therefore, it was difficult to predict recurrence after radical resection of CRC by perioperative *BRAF* V600E.

This study showed that the MAF of *BRAF* V600E did not correlate with recurrence or tumor markers or lymph node metastasis, but with tumor diameter. Bando et al. compared the concordance rate of RAS mutations between cfDNA in plasma and tumor tissue DNA in patients with metastatic CRC^31^. They reported that patients with pulmonary metastases only, with a maximum lesion diameter of 20 mm and fewer than 10 lesions, had less tumor released into the plasma, which decreased the detection sensitivity of cfDNA. Because the detection rate varies depending on the site of metastasis, tumor diameter, and number of metastases, it may be difficult to predict recurrence using cfDNA in the early postoperative period.

Early studies established that many cancer cells are disseminated into the blood from an early stage, but metastasis is a highly inefficient process^32–34^. These studies showed that within 24 h after entry into the circulation, less than 0.1% of tumor cells are still viable, and less than 0.01% of these cells, when introduced into the circulation, survive to produce metastases. Therefore, only a few cells in a primary tumor can give rise to a metastasis.

We were able to collect samples from two recurrent cases. In these cases, the MAF of *BRAF* V600E mutation had increased over time. This may be due to the increased amount of tumor in the body caused by the recurrence. Although recurrence could not be predicted from cfDNA in the perioperative period, it may be possible to detect recurrence early by measuring it in succession. If the detection sensitivity of liquid biopsy is improved by further research and the accumulation of knowledge, *BRAF* mutated cases may be detected earlier, permitting earlier therapeutic intervention.

The present study has several limitations. First, it was conducted at a single institution. Second, only 3.7% of patients had *BRAF* V600E, which was a small number of eligible patients. Third, because the observation period of our study was 22 months, it is possible that other patients may relapse in the future. Finally, we analyzed only *BRAF* V600E by dPCR. Cancer is spatially and temporally heterogeneous, and recurrent tumors may not have the *BRAF* V600E mutation. In the future, other genetic mutations should be studied.

In conclusion, it is difficult to predict early recurrence by measuring cfDNA for *BRAF* V600E in the perioperative period. Metastasis is known to be a very inefficient process, and this may make an early prediction of recurrence difficult.

## Methods

### Study design and patients

This was a prospective study that included patients who underwent colorectal surgery without distant metastasis at Fukuoka University Hospital between April 2018 and March 2020. A total of 362 patients diagnosed with primary colorectal adenocarcinoma who underwent primary tumor resection were included. For tissue *KRAS*, *NRAS*, and *BRAF* tests, the MEBGEN RASKET-B kit (MBL, Tokyo, Japan), which applies the polymerase chain reaction-reverse sequence-specific oligonucleotide method, was used in accordance with the manufacturer’s protocol^35^. Cases with wild type and *KRAS*/*NRAS* mutations were excluded. The institutional review board of Fukuoka University Faculty of Medicine approved this study (2017M35, U19-09-001). Written informed consent was obtained from all patients. All procedures were performed in accordance with the Declaration of Helsinki.

### Blood collection procedures

Peripheral blood was collected within 1 week before surgery and on postoperative days 1 and 30. For each patient, 10 ml blood was collected in a BD Vacutainer® PPT plasma preparation tube (Becton, Dickinson and Company, Franklin Lakes, NJ, USA). The sample was centrifuged at 1100×*g* for 10 min at 4 °C within 2 h after blood collection. The plasma was transferred to a microtube and stored at − 80 °C until use.

### cfDNA extraction from frozen plasma samples

Plasma samples kept at − 80 °C were re-centrifuged at 16,000×*g* for 10 min at 4 °C to eliminate debris. cfDNA was extracted from 1 ml plasma using the Maxwell® RSC cfDNA plasma kit (Promega Corporation, Madison, USA) and Maxwell® RSC Instrument (Promega Corporation, Madison, USA) in accordance with the manufacturer’s protocol, as described previously^36^.

### Mutation detection by dPCR and sequencing data analysis

The quantity of cfDNA was calculated using the QuantStudio™ 3D Digital PCR System (Applied Biosystems, South San Francisco, CA)^37^. Each polymerase chain reaction (PCR) mixture was prepared with 9 μL QuantStudio 3D Digital PCR Master Mix, 0.45 μL TaqMan™ assays, and 8.1 μL cfDNA. We loaded 15 μL of the 17.1 μL reaction mixture onto a QuantStudio™ 3D Digital PCR 20 K Chip using the automatic chip loader. The DNA amplification reaction using the ProFlex™ PCR System (Applied Biosystems, South San Francisco, CA) was 96 °C for 10 min followed by 39 cycles of 56 °C for 2 min and 98 °C for 30 min, and 39 cycles at 56 °C for 2 min and 98 °C for 30 s, with a final extension step at 60 °C for 2 min. For dPCR, predesigned dual-probe TaqMan assays were purchased from ThermoScientific for *BRAF* V600E (c.1799T>A; Assay Hs000000004_rm). Results were analyzed using QuantStudio 3D Analysis Suite™ Cloud software. Automatic call assignment for each data cluster was manually adjusted as needed. dPCR data analysis was performed by two independent investigators blinded to clinical and tumor information. The results of the assay are reported as MAF, defined as the ratio of mutant DNA molecules to the sum of wild-type and mutant DNA molecules. A sample was considered positive when the MAF was greater than 0.15%^22^.

Plasmid DNA harboring the *BRAF* V600E mutation (GeneArt, Thermo Fisher Scientific) was used to confirm the sensitivity of dPCR for the *BRAF* V600E mutation. We generated plasmid dilutions down to 0.1% *BRAF* V600E mutation on a wild-type plasmid DNA background and used the QuantStudio™ 3D dPCR System to quantify MAF and plasmid copy numbers. MAF determined by dPCR was in high concordance compared with the allele concentration built by plasmid constructs (R^2^ = 0.99, p = 0.003). dPCR detected the presence of the *BRAF* V600E mutation in all dilutions down to 0.1%.

### Statistical analysis

Statistical analysis was conducted using IBM SPSS Statistics (IBM Japan, Inc., Tokyo, Japan). Results are presented as number or median (interquartile range [IQR]). We performed comparisons using the Mann–Whitney U test for quantitative variables. *P*-values of < 0.05 were considered significant.

## Data Availability

The datasets generated during and/or analyzed during the current study are available by request.
